# Effects of Metformin on Mitochondrial Health and Oxidative Stress in Age-Related Macular Degeneration: A Systematic Review

**DOI:** 10.7759/cureus.104820

**Published:** 2026-03-07

**Authors:** Raghavee Neupane, Jami Rance, Tara McKenna, Marc M Kesselman

**Affiliations:** 1 Medicine, Nova Southeastern University, Dr. Kiran C. Patel College of Osteopathic Medicine, Fort Lauderdale, USA; 2 Rheumatology, Nova Southeastern University, Dr. Kiran C. Patel College of Osteopathic Medicine, Fort Lauderdale, USA

**Keywords:** age-related macular degeneration, cellular senescence, longevity, metformin, mitochondrial function, telomere maintenance

## Abstract

Age-related macular degeneration (AMD) is a chronic, progressive condition and a leading cause of irreversible central vision loss in older patients. It is driven by oxidative stress, mitochondrial dysfunction, chronic inflammation, and degeneration of retinal pigment epithelium (RPE) cells. Current AMD treatments include lifestyle modifications, nutritional supplements, and/or anti-vascular endothelial growth factor therapies and primarily aim to slow disease progression. As a result, interest has grown in repurposing established medications with potential cytoprotective properties. Metformin, a widely-used anti-diabetic agent, has been proposed as a candidate due to its anti-inflammatory and mitochondrial-modulating effects. We conducted a systematic review to identify studies published between January 2015 and November 2025 that evaluate metformin therapy in (1) adults with AMD and (2) experimental retinal models designed to replicate AMD-related degeneration or pathogenesis. Comparators included individuals with AMD who were not taking metformin therapy, were untreated, and/or were given standard treatment therapy. Outcomes of interest included in the review focused on clinical endpoints of AMD incidence and severity and mechanistic endpoints of mitochondrial function, oxidative stress markers, and cellular senescence. This systematic review includes evidence from epidemiologic, clinical, and experimental studies to link molecular mechanisms with observed disease progression. A total of 10 studies published met the inclusion criteria and demonstrated that metformin is associated with cytoprotective effects in RPE cells by reducing oxidative stress (ROS) and upregulating antioxidant enzymes through activation of the Nrf2 pathway. The drug was also shown to preserve mitochondrial function via activation of AMP-activated protein kinase by enhancing mitophagy, supporting DNA repair, and promoting mitochondrial biogenesis. Observational studies suggested that metformin use was associated with a lower risk of AMD development, particularly dry AMD, with stronger associations observed with a longer duration and higher cumulative exposure. However, findings were context-dependent. Under certain stress conditions, such as sodium iodate exposure, metformin-mediated inhibition of mitochondrial complex I appeared to increase oxidative stress, highlighting a potential "double-edged" effect. Overall, current preclinical and observational studies suggest a possible association between metformin use and mitochondrial modulation in AMD. Prospective studies are needed to clarify dosing, safety, and therapeutic relevance before clinical recommendations can be made.

## Introduction and background

Aging is marked by progressive cellular dysfunction, including mitochondrial impairment, oxidative stress, and telomere shortening. Mitochondrial health and telomere integrity are recognized hallmarks of cellular aging and contribute to cellular senescence and increased susceptibility to age-related degenerative diseases [1]. Age-related macular degeneration (AMD) is a leading cause of irreversible vision loss worldwide and represents a significant public health burden in the aging population, particularly among adults over 60 years of age [2].

AMD exists in two forms: dry (atrophic), which accounts for 80-90% of cases, and wet (neovascular or exudative). Dry AMD is characterized by the accumulation of extracellular deposits called drusen and the progressive loss of retinal pigment epithelium (RPE) cells, photoreceptors, and choroidal capillaries. In RPE cells, whose large energy requirements and exposure to ongoing oxidative damage increase with age and are thought to be a primary contributor to ADM pathogenesis [2,3]. Oxidative damage increases with age and is thought to be a primary contributor to AMD pathogenesis through protein, lipid, DNA, and mitochondrial damage, and disease progression. Current treatment aims to slow the progression of disease activity, rather than restoring vision that has already been lost [2,3]. In neovascular AMD, elevated levels of vascular endothelial growth factor (VEGF) stimulate the growth of abnormal vasculature and increase vascular leakage [3]. Anti-VEGF medications work by blocking this signal, helping to reduce new vessel formation and fluid accumulation, which can stabilize, and in many cases, improve vision [3]. Anti-VEGF therapies have been shown to significantly improve visual outcomes in patients with wet AMD [2] but require frequent treatments. For patients with dry AMD, anti-VEGF therapies do not address mitochondrial dysfunction, thought to drive disease progression [2]. Further, patients with dry AMD receive little to no benefit from these therapies due to the progressive atrophy of RPE cells [2,3]. 

Metformin, a biguanide antihyperglycemic agent, remains the most widely prescribed first-line treatment for type 2 diabetes mellitus since its approval by the United States Food and Drug Administration (FDA) in 1994 [4-6]. Although metformin is not an anti-VEGF agent, preclinical and clinical research have shown that metformin is associated with improvements in mitochondrial function, a marker of cellular health and age-related decline [5,7-9]. It acts on hallmarks of aging, through activation of AMPK, inhibition of mitochondrial complex I (NADH: ubiquinone oxidoreductase), enhanced autophagy, reduced oxidative stress, and improved mitochondrial biogenesis and efficiency [9].

Metformin has also been proposed to influence telomere biology in broader aging contexts. Experimental and epidemiologic studies outside the AMD field suggest that metformin may attenuate telomere shortening through reductions in oxidative stress and improvements in cellular metabolic regulation [4,6,7]. However, direct evidence linking metformin to telomere preservation specifically in RPE cells or in the context of AMD remains limited. Telomere-related mechanisms in AMD should be considered theoretical and exploratory rather than definitively established.

Because mitochondrial dysfunction and cellular aging play key roles in the development of AMD, it is important to understand whether metformin affects these processes in retinal tissue. This review synthesizes current evidence on metformin's reported effects on mitochondrial function in the context of AMD. The aim of the study is to conduct a systematic review that integrates both clinical and preclinical evidence to provide a mechanistic perspective on the potential role of metformin in modifying age-related retinal degeneration.

Review question

Does metformin improve mitochondrial function and/or telomere integrity, and can these effects slow the progression of AMD? To systematically define the scope of this review, we formulated the research question using the Population, Intervention, Comparison, and Outcomes (PICO) framework (Table 1).

**Table 1:** PICO Framework for Evaluating Metformin in Age-Related Macular Degeneration Experimental models of AMD, including retinal cell cultures, iPSC-derived RPE lines, and animal models investigating mitochondrial aging mechanisms related to AMD pathology. AMD: Age-related macular degeneration; RPE: retinal pigment epithelium; iPSC: induced pluripotent stem cell

Population (P)	Adults (>18 years old) diagnosed with dry or wet AMD or experimental models of AMD (including retinal cell cultures, iPSC-derived RPE lines, and animal models) investigating mitochondrial aging mechanisms related to AMD pathology.
Intervention (I)	Clinical metformin use or metformin cellular exposure
Comparison (C)	No metformin exposure, alternative metabolic/AMPK-modulating agents, no treatment or standard therapies (anti-VEGF agents)
Outcomes (O)	Mitochondrial function, oxidative stress markers, telomere integrity, cellular senescence, retinal cell viability, and progression or risk of AMD

## Review

Methods

Search Strategy

A comprehensive literature search was conducted to identify studies evaluating the effects of metformin on mitochondrial function, telomere integrity, and age-related disease progression in adults with AMD. Searches were performed using Embase, Web of Science, and OVID MEDLINE (January 1, 2015 - November 15, 2025). Search terms included combinations of controlled vocabulary and keywords related to the condition (i.e., "macular degeneration"[MeSH Terms] OR AMD[Title/Abstract] OR "retinal pigment epithelium"[MeSH Terms] OR RPE[Title/Abstract]), intervention (i.e., [metformin[Title/Abstract] OR biguanides[MeSH Terms]), and outcomes (i.e., mitochondria[Title/Abstract] OR "mitochondrial function"[Title/Abstract] OR "mitochondrial dysfunction"[MeSH Terms] OR mitophagy[Title/Abstract] OR "oxidative stress"[MeSH Terms] OR Nrf2[Title/Abstract] OR "telomere shortening"[Title/Abstract] OR telomerase[MeSH Terms] OR "cellular senescence"[MeSH Terms]). Boolean operators (“AND,” “OR”), truncation, and database-specific filters were applied to refine the search. This review protocol was not prospectively registered (e.g., in PROSPERO); however, the methodology was predefined by the authors prior to study selection and data extraction.

Inclusion Criteria

Studies were eligible for inclusion if they focused on the investigative effects of metformin on AMD or AMD-related retinal degeneration cell models. The review aimed to incorporate both clinical studies involving adults diagnosed with AMD or in-vitro studies replicating AMD retinal disease (e.g., RPE cell lines, iPSC-derived RPE models). To be eligible, studies had to report on the effects of metformin on mitochondrial function, including mitochondrial biogenesis, oxidative stress, mitochondrial dysfunction, telomere integrity, and cellular senescence. Relevant outcomes included clinical measures of aging, mitochondrial markers, telomere length, or markers of age-related disease progression. The review considered primary research studies, including randomized controlled trials (RCTs), cohort studies, observational studies, case-control studies, experimental studies, and case reports. Only studies published in English between 2015 and 2025 were included.

Exclusion Criteria

Studies were excluded if they involved non-human subjects, including animal studies. In vitro studies using human retinal pigment epithelial models were included due to clinical relevance of AMD pathogenesis. Studies that did not focus on adult populations with AMD or similar retinal conditions were excluded. In addition, studies were excluded if they did not report relevant outcomes related to mitochondrial function, telomere integrity, or age-related disease markers. Review articles, commentaries, editorials, protocols, and conference abstracts without full text were also excluded. Studies that lacked adequate methodological detail, such as unclear interventions, outcomes, or poor study quality (i.e., no control groups or small sample sizes), were excluded. In addition, studies that evaluated interventions other than metformin (i.e., diabetes treatments not involving biguanides) were excluded from the review. Disagreements during screening were resolved by a 3rd reviewer and through discussion.

Data Extraction

All identified records were screened using a two-stage process. Titles and abstracts were first reviewed for relevance to the research question. Full-text screening was then performed for articles meeting preliminary criteria. Authors (RN, TM) independently assessed the eligibility criteria of each article. A 3rd researcher (JR) resolved discrepancies.

Study Selection and Critical Appraisal of the Evidence

**Figure 1:**
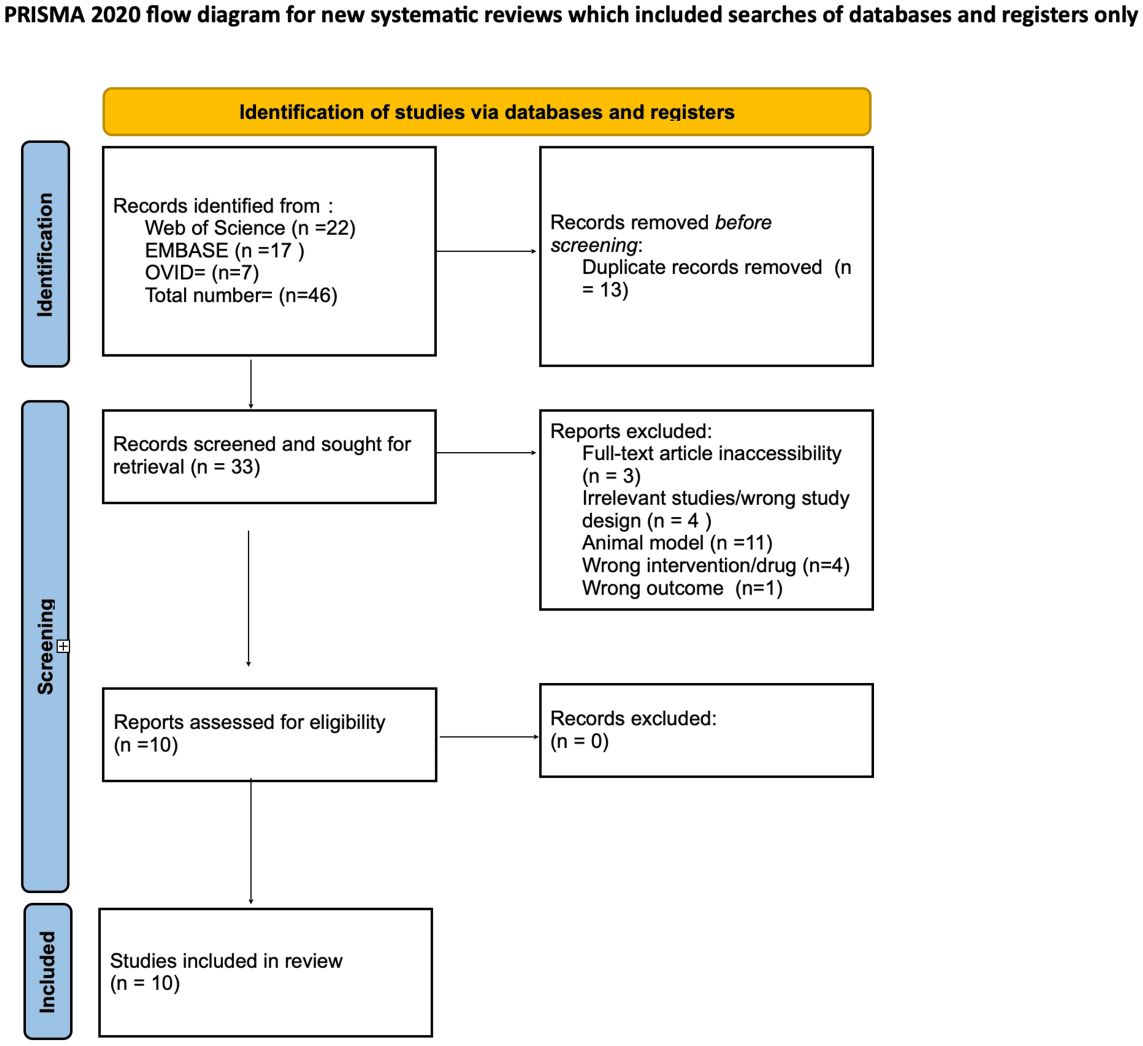
Preferred Reporting Items for Systematic Reviews and Meta-Analyses (PRISMA) flow diagram illustrating the study selection process

The search yielded 46 studies, of which, after removing 13 duplicates, 33 unique articles remained. Title and abstract screening (Tier 1) by two independent reviewers reduced this to 11 full-text articles (Tier 2), of which 10 met the inclusion criteria. Quality assessment using the Joanna Briggs Institute critical appraisal tool categorized the risk of bias as low (>70%), moderate (50%-70%), or high (<50%) [10]. Risk-of-bias ratings were considered when interpreting findings, with higher-quality studies weighted more heavily in the narrative synthesis to support our conclusions. Two blinded reviewers assessed each study, resolving disagreements through discussion or consultation with a third reviewer. All included studies were classified as having a low risk of bias. The selection process is illustrated in the PRISMA-ScR flow diagram (Figure 1).

Results

Among the studies included in this review, metformin demonstrated cytoprotective effects on RPE cells, primarily through attenuation of oxidative stress, modulation of mitochondrial function, and activation of key metabolic signaling pathways. Exposure of ARPE-19 cells to hydrogen peroxide (H₂O₂) induced significant reductions in cell viability, nuclear damage, and elevated secretion of pro-inflammatory cytokines, including IL-1β, IL-6, TNF-α, and IL-8 [11]. Pretreatment with metformin (0.5-5 mM, concentrations above those achievable in human plasma) mitigated the inflammatory effects in a dose-dependent manner, restoring cell viability, reducing reactive oxygen species (ROS), enhancing glutathione levels, and upregulating antioxidant enzymes such as SOD2 and catalase [11]. Mechanistic studies revealed that metformin activated the Nrf2 signaling pathway, leading to upregulation of the transcription of antioxidant genes, including HO-1 and NQO1. Genetic knockout of the Nrf2 gene abolished the cytoprotective effects of metformin, indicating that Nrf2 activation is essential for the antioxidant and anti-inflammatory actions exhibited in RPE cells [11].

In addition to antioxidant activity, metformin preserved mitochondrial function, a key factor in AMD pathogenesis. Nutrient starvation of ARPE-19 cells led to mitochondrial fragmentation, increased ROS, and reduced AMPK phosphorylation, resulting in cellular senescence [12]. Treatment with metformin or pharmacological AMPK activators restored mitochondrial morphology, enhanced mitochondrial biogenesis via PGC1-α and TFAM upregulation, suppressed NF-κB signaling, and limited epithelial-to-mesenchymal transition (EMT) [12]. In iPSC-derived RPE cells from patients with AMD, mitochondrial respiration and AT production were impaired, and mitochondrial morphology was abnormal. Treatment with metformin partially reversed these effects and preserved RPE-specific markers such as PEDF and VEGF [13]. These findings suggest that AMPK activation mediates the beneficial effects of metformin on mitochondrial integrity and cellular homeostasis.

Metformin displays enhancement of mitochondrial quality control mechanisms, including mitophagy and mitochondrial DNA repair. In a study, metformin protected retinal pigment epithelial cells from hydrogen peroxide-induced oxidative stress by activating AMPK-dependent autophagy. Metformin treatment lowered reactive oxygen species levels, preserved mitochondrial membrane potential, reduced apoptosis, and improved overall cell viability. When autophagy or AMPK signaling was blocked using pharmacologic inhibitors or gene silencing, these protective effects were lost. Together, these findings indicate that metformin exerts its protective effects in RPE cells primarily by enhancing autophagic flux through activation of the AMPK pathway [14]. In other experimental RPE cell models, metformin treatment promoted mitophagy, reduced secretion of inflammatory cytokines (IL-6, IL-8), and mitigated ROS-mediated injury [15]. This highlights metformin's role in maintaining mitochondrial quality and cellular homeostasis. Collectively, these findings encourage the understanding that metformin acts in maintaining mitochondrial morphology and preserves cellular homeostasis while under conditions of stress.

Despite metformin's cytoprotective effects, its actions displayed differing effects depending on the stressor used. In ARPE-19 cells exposed to sodium iodate (NaIO₃), metformin exacerbated cytotoxicity by inhibiting mitochondrial complex I, whereas A769662, a selective AMPK activator, preserved mitochondrial respiration and morphology [16]. Alternatively, under UVA-induced oxidative stress, metformin and A769662 both reduced mitochondrial ROS production and autophagy-dependent cell death. While AMPK activation has been shown to be cytoprotective in RPE cells, metformin has been found to either aggravate or attenuate the effects of oxidative damage depending on the type of oxidative or metabolic stress implemented [17].

Clinical and Epidemiologic Evidence for Metformin in AMD

Observational clinical studies suggest that metformin use is potentially associated with a lower risk of developing AMD. As these studies are observational, causality cannot be inferred, and confounding factors such as diabetes severity, comorbidities, or concurrent medications may influence outcomes. A retrospective cohort study of 68,205 Taiwanese patients with type 2 diabetes found that metformin users had a lower cumulative incidence of AMD (3.4%) compared with nonusers (5.6%), with evidence of dose- and duration-dependent associations [18]. These associations remained significant after adjusting for age, sex, and comorbidities, suggesting a protective effect independent of baseline health status.

Similarly, a U.S.-based nested case-control study reported that metformin use was associated with reduced odds of AMD, independent of diabetes status and other confounding factors [19]. In this cohort of 7,788 adults aged ≥55 years, patients on metformin were less likely to develop AMD compared with nonusers, while other antidiabetic medications, including DPP-4 inhibitors and statins, did not demonstrate comparable associations. Analysis of a U.S. insurance claims database examining dry AMD suggested modestly decreased risk with prior metformin use, though associations varied by dose, timing, and duration of exposure [20].

Overall, these epidemiologic data align with mechanistic evidence from in vitro studies, suggesting that metformin can serve to preserve RPE integrity through antioxidant defense and provide mitochondrial protection and AMPK activation, as well as promote mitophagy. Although causality cannot be established from observational studies, the reproducibility across studies and dose/duration trends support further exploration of metformin as a potential preventive or therapeutic agent for AMD. Table 2 summarizes the included studies, detailing the authors/year, study design, intervention, outcomes, key results, limitations, and risk of bias (JBI).

**Table 2:** Impact of Metformin on AMD Progression: Summary of Evidence and Risk-of-Bias Evaluation A769662: Direct adenosine monophosphate-activated protein kinase (AMPK) activator; AICAR: 5-Aminoimidazole-4-carboxamide ribonucleotide (AMPK activator); Akt: Protein kinase B; AMPK: AMP-activated protein kinase; ATG5: Autophagy-related 5; BR: Basal respiration; CAP: Chloramphenicol; CAT: Catalase; CCI: Charlson Comorbidity Index; CCK-8: Cell Counting Kit-8; CMST: Cell migration and signaling test; DCF-DA: 2’,7’-Dichlorodihydrofluorescein diacetate; DHE: Dihydroethidium; DPI: Diphenyleneiodonium; DPP-4: Dipeptidyl peptidase-4; Drp1: Dynamin-related protein 1; EMT: Epithelial–mesenchymal transition; ERK: extracellular signal-regulated kinase; GSH: Glutathione; HBSS: Hanks’ balanced salt solution; HO-1: Heme oxygenase-1; iPSC-RPE: Induced pluripotent stem cell–derived retinal pigment epithelium; JC-1: Mitochondrial membrane potential dye; LC3 (LC3-I/LC3-II): Microtubule-associated protein 1 light chain 3; MAP1LC3B: Microtubule-associated protein 1 light chain 3 beta; MR: Maximal respiration; MTT: Methylthiazolyldiphenyl-tetrazolium bromide; NAC: N-acetylcysteine; NaIO₃: Sodium iodate; NQO1: NAD(P)H quinone dehydrogenase 1; Nrf2: Nuclear factor erythroid 2-related factor 2; OCR: Oxygen consumption rate; PARP-1: Poly(adenosine diphosphate-ribose) polymerase 1; PEDF: Pigment epithelium–derived factor; PGC1-α: Peroxisome proliferator-activated receptor gamma coactivator 1-alpha; SNAI1: Snail family transcriptional repressor 1; SOD1: Superoxide dismutase 1; SQSTM1/p62: Sequestosome 1; SRC: Spare respiratory capacity; TCA: Tricyclic antidepressant; TFAM: Mitochondrial transcription factor A; TFEB: Transcription factor EB; TOM20: Translocase of outer mitochondrial membrane 20; U0126: Mitogen-activated protein kinase/extracellular signal-regulated kinase pathway inhibitor; γ-H2AX: Phosphorylated histone H2AX

Authors/Year	Study Design	Intervention	Outcome	Results Summary	Limitations	Risk of Bias (JBI)
Feng et al. (2024) [11]	Experimental, mechanistic in vitro study	0.5-5 mM metformin was administered to ARPE-19 RPE cells. Nrf2 knockout via CRISPR/Cas9 to test whether metformin’s effects depend on Nrf2 activation.	1. Oxidative stress (glutathione redox status & antioxidant enzyme expression- SOD1, CAT, HO-1, NQO1). 2. Inflammatory cytokines (IL-6, IL-1beta, TNF-alpha, IL-8). 3. Nrf2 expression. 4. Cell viability (CCK-8 assay) and cell morphology (Hoechst staining)	1. Metformin significantly reduces H_2_O_2_-induced cell death. 2. ROS levels decreased by ~32% at 2mM metformin, restoration of antioxidant and improved GSH levels. 3. Marked reduction in pro-inflammatory cytokines in a dose-dependent manner. 4. Knockout Nrf2 samples showed no benefit, confirming that metformin requires the Nrf2 gene to perform protective factors in RPE cells.	1. ARPE-19 retinal cells are transformed; therefore, they may not directly reflect a human RPE. 2. The Nrf2 pathway and mechanism are unclear. 3. Metformin (0.5-5 mM) may exceed clinically achievable plasma levels through oral dosing in patients.	Low
Park et al. (2023) [12]	Controlled in vitro experimental study	Metformin (1 mM) and AICAR for AMPK Activation, Compound C for AMPK Inhibition, Chloramphenicol (CAP) for mitochondrial biogenesis inhibition, Hanks’ Balanced Salt Solution (HBSS) media for 0-36 hours for nutrient starvation	1. Oxidative Stress: ROS via DCF-DA fluorescence 2. Mitochondrial Health: AMPK phosphorylation, MitoTimer, MitoTracker, membrane potential, PGC1-a nuclear translocation, TFAM expression and Morphology (Confocal microscopy, TEM) 3. EMT markers: E-Cadherin (CDH1), Vimentin (VIM), TWIST1, SNAI1 4. Inflammatory Response (NF-kB activation (luciferase))	1. Starvation increases ROS & mitochondrial senescence; AMPK decreases at 12 h. 2. AMPK activation (Met/AICAR): decreases ROS, senescence, NF-KB/TWIST1, EMT (lower VIM/CDH1), and increases PGC1-a/TFAM. 3. AMPK inhibition (Comp C/CAP): Decreases biogenesis, increases ROS and EMT. 4. Mechanism: AMPK activates PGC1-α → TFAM promotes mitochondrial renewal, while lowering ROS blocks NF-κB → TWIST1 signaling to prevent EMT.	1. ARPE-19 RPE cell line only. 2. HBSS Starvation may not mimic aging/AMD metabolism. 3. Short-term duration (0–36 h). 4. Metformin 1 mM = supraphysiologic. 5. Other retinal layers and EMT pathways have not been fully explored. 6. Comp C and CAP may have non-specific effects beyond AMPK	Low
Ebeling et al. (2022) [13]	Controlled in-vitro experimental study	1. AMD patient iPSC-RPE treated with AICAR, Metformin (AMPK activators), or Trehalose (autophagy enhancer). 2. Short-term (48 h) & long-term (3 wk) exposures. 3. Mitochondrial function measured via CMST, ATP assays, and RPE functional tests.	1. Mitochondrial respiration: BR, MR, SRC, ATP-Linked Respiration. 2. ATP production: mitochondrial + glycolytic. 3. RPE function: PEDF/VEGF secretion, phagocytosis, mitochondrial morphology/protein expression.	1. AMD iPSC-RPE shows mitochondrial dysfunction; Those cells treated with AICAR, metformin, and trehalose improve BR, MR, SRC, ATP-linked respiration, ATP production, and RPE function 2. ~8–10% variability between patient lines. 3. iPSC-RPE models recapitulate AMD defects and support personalized drug screening	1. Small sample (n=5) limits generalizability. 2. Preventive effects are unknown since patients already had AMD. 3. Genetic heterogeneity may affect response. 4. iPSC-RPE may not fully replicate the in vivo environment. 5. Some functional assays (e.g., autophagy, ROS) were not measured for all drugs.	Low
Zhao et al. (2020) [14]	Controlled in vitro experimental study	1) Metformin (0.125-2 mM; primarily 1 mM). 2) ± H202 (200 M). 3) Autophagy inhibitors: 3-MA, chloroquine. 4) ShRNA knockdown: Beclin1, LC3B, AMPK. 5) AMPK inhibitor: Compound C	1) Cell viability (MTT, LDH). 2) Apoptosis (TUNEL, Hoechst, flow cytometry). 3) ROS levels. 4) Mitochondrial membrane potential (JC-1). 5) Autophagy markers (LC3-II, p62, P-ULK1, PBeclin1). 6) AMPK phosphorylation	1) Metformin protected RPE cells from H₂O₂-induced oxidative stress. 2) Reduced ROS and maintained mitochondrial membrane potential. 3) Enhanced autophagic flux (↑LC3-II, ↓p62) via AMPK activation (↑p-AMPK, ↑ULK1 & Beclin1 phosphorylation). 4) Inhibition of autophagy or AMPK abolished protective effects. 5) Similar protective effects observed in primary human RPE cells.	1) Fully in vitro; no animal or human AMD model. 2) Acute H202 stress may not mimic chronic AMD. 3) Metformin dose (1 mM) exceeds typical plasma levels. 4) No in vivo pharmacokinetics or dose translation. 5) Focused on AMPK; other pathways not assessed	Low
Toppila et al. (2024) [15]	Controlled in vitro experimental study	1) Human RPE-derived cells (ARPE-19). 2) Metformin pretreatment for one hour before exposure to mitochondrial toxin Antimycin A (10 µM)	1) Cell viability: MTT assay (24h exposure to 10-40mM Metformin; LDH-release assay). 2) mtROS measured by MitoSOX red superoxide indicator and Total cellular ROS measured by H2DCFDA fluorescence). 3) % changes of IL-6 and IL-8 4) Measures autophagy by a) Tracking key proteins: SQSTM1/p62 and LC3-I/II (Western blot/immunochemistry), TOM20 mitochondrial outer membrane marker (immunofluorescence)) and b) gene expression (SQSTM1, MAP1LC3B, MTOR)	1) Metformin reduced ROS and improved ARPE-19 cells viability. 2) IL-6 and IL-8 production decreased to 42% and 65% in cells with Metformin pretreatment. 3) Mitophagy/autophagy markers increased in metformin-treated cells	1) ARPE-19 cell line = limited in vivo relevance. 2) Acute Antimycin A damage may not model chronic/physiological stress. 3) Only short-term biochemical/cellular markers assessed; no long-term visual outcomes	Low
Chan et al. (2020) [16]	Controlled in-vitro experimental study	1. A769662 (selective AMPK activator): Pretreatment of ARPE-19 cells before NaIO₃. 2. Metformin: AMPK activator + mitochondrial complex I inhibitor; at multiple concentrations. 3. Comparators/mechanistic probes a) NAC, Trolox (antioxidants), b) DPI (NADPH oxidase inhibitor), c) Rotenone (complex I inhibitor), and d) U0126 (MEK/ERK inhibitor)	1. Cell health: viability (Annexin V/PI), DNA damage (γ-H2AX). 2. ROS: cytosolic (DCFDA, DHE), mitochondrial (MitoSOX, MitoPY1). 3. Mitochondria: membrane potential (JC-1, Rhodamine 123), respiration/OCR (Seahorse), fission & mass (Tom20, Mitotracker). 4. Signaling: Drp1, ERK, Akt phosphorylation (Western blot)	1. A769662 (Beneficial) a. Protects RPE from NaIO₃ by restoring ATP + cellular respiration; b. reduces fission and ERK/Akt-Drp1(S616) phosphorylation; c. protects against ROS independently; 2. Metformin (Harmful) a. Worsens NaIO₃-induced death; b. fails to restore ATP/respiration; c. does not block fission or Drp1 phosphorylation; d. toxicity likely via complex I inhibition (similar to rotenone).	1. In vitro (ARPE-19 cell line; limited in-vivo relevance). 2. High NalOs, NAC, and metformin concentrations may exceed physiological ranges. 3. No direct AMPK inhibition to confirm mechanism. 4. Single cell line model without RPE or animal validation. 5. Potential off-target effects of pharmacologic tools (e.g., A769662, U0126, DPI). 6. Limited temporal resolution; short-term signaling effects may be missed.	Low
Wu et al. (2023) [17]	Controlled in-vitro experimental study	1) Model: Human RPE-derived cells (ARPE-19) exposed to UVA. 2) Pretreatment with either a) ROS scavengers (N-acetylcysteine 5mM or mitoTEMPO 100µM), b) autophagy inhibitors (3-methyladenine 5mM), c) gene silencing via siRNA against ATG5 (testing autophagy) or d) AMPK activation (A769662 (25µM) or Metformin (> 6mM))	1) Cell viability: Annexin V-FITC/PI Flow cytometry (18 h post-UVA). 2) mtROS using MitoSOX Red) and Total cellular ROS (1 h post-UVA). 3) Autophagy assessed by LC3-I/II and p62 protein levels at 0.5, 1, 3, and 6 h post-UVA (Western blot), TFEB mRNA expression (RT-qPCR 5 h post-UVA). 4) Mitochondrial function: mitochondrial membrane potential (JC-1 stain 1 h post-UVA), oxygen consumption (Seahorse XF flux analyzer) 5) DNA damage via activation of PARP-1 (immunoblot) 6) Lysosomal function: lysosomal mass and activity of cathepsin B	1) UVA induced mitochondrial ROS within 1h. 2) ARPE-19 cells showed 50% viability loss 18h post-UVA. 3) Increased LC3-II, p62 (6h), and upregulation of TFEB by 5h. 4) Pretreatment with Metformin/A769662 reduced UVA-induced cell death, mtROS, LC3 puncta/LC3-II and reversed mitochondrial fission. 5) AMPK activation suppressed PARP-1 signaling. 6) Decreased lysosomal mass and cathepsin B activity during mid-to-late phase after UVA	1) The ARPE-19 cell line does not fully reflect in vivo retinal tissue complexity. 2) Acute, high-dose UVA may not reflect chronic physiologic light exposure. 3) Supraphysiologic metformin concentrations (>6 mM) limit clinical relevance. 4) Long-term visual functional outcomes were not assessed. 5) Mechanisms underlying lysosomal impairment are unclear	Low
Chen et al. (2019) [18]	Retrospective cohort study	Metformin users (n=45,524); non-metformin users (n=22,681)	1. Development of AMD confounding variables adjusted for: age, sex, HTN, hyperlipidemia, CAD, obesity, diabetic retinopathy, CKD, and Insulin use - used propensity score	1. Metformin users had a significantly lower risk of AMD - Adjusted HR = 0.54 (46% lower risk) - Propensity score match HR = 0.57 (43% lower risk). 2. Longer metformin therapy correlated with greater risk reduction a) 1.5-4 years HR= 0.52 and b) >4 years HR= 0.14 = 86% risk reduction. 3. Higher metformin dose was associated with lower AMD risk - Moderate dose: HR = 0.59 - High dose: HR = 0.27 (p-value < 0.05)	1. Observational study. 2. Residual confounding. 3. Missing lifestyle/UV exposure data. 4. No lab values (A1c & GFR) included. 5. Medication adherence unknown	Low
Brown et al. (2019) [19]	Retrospective case-control study	Cases: 1,947 AMD patients (≥4 visits before diagnosis). Controls: 5,841 non-AMD patients, 3:1 matched by age, CCI, HTN, and anemia. Exposure: History of metformin use	Odds of developing AMD w/ metformin use vs co-variety Covariates: age, sex. BMI, race/ethnicity, clinical comorbidities, ocular diagnoses, medication use (statins, antidepressants, DPP-4 inhibitors)	1. Metformin lowers AMD risk - Univariate OR: 0.39 (95% CI: 0.31–0.49) - Multivariable OR: 0.58 (95% CI: 0.43–0.79). 2. DPP-4 inhibitors and statins → No significant effect after adjustment - SSRIs and TCA → INCREASED risk of AMD. 3. Diabetic subgroup: Metformin still protective - Multivariable OR: 0.70 (95% CI: 0.49–0.98)	1. Observational study. 2. Unspecified AMD (wet vs dry). 3. Missing risk factor data could lead to potential residual confounding. 4. Medication adherence unknown	Low
Eton et al. (2022) [20]	Retrospective cohort study using a large US insurance claim database	Adults >55 y: Dry AMD cases vs. non-AMD controls. Exposure: Metformin use from prescription records. Covariates adjusted in analysis.	1. Primary outcome = Development of dry AMD w/ and w/o Metformin use. 2. Secondary outcome = Protective effects of other therapeutics on AMD	1. Metformin significantly reduced the odds of developing dry AMD - Adjusted OR ~0.58 (42% risk reduction). 2. Diabetic subgroup w/ metformin: - OR ~0.70. 3. Other therapies do not have a protective effect, SSRIs/Tetracyclines increased dry AMD risk. 4. Known AMD risk trends were higher in men	1. Observational study only. 2. Metformin dose/duration not reported. 3. Lacks lifestyle data (i.e. diet, exercise, etc.)	Low

Discussion

Effective disease-modifying therapies remain limited for AMD, particularly for the non-exclusive form. The studies included in this review consistently demonstrate that metformin exerts multi-faceted cytoprotective effects on known pathogenic mechanisms of AMD. These findings suggest that metformin modulates oxidative stress responses [11,12,15,16], preserves mitochondrial quality [12-14,17], regulates metabolic signaling pathways [12,16], and improves long-term retinal outcomes [18,19,20], displaying its promising candidacy as a therapeutic agent and slowing disease progression.

In vitro models demonstrated that metformin consistently reduced ROS, preserved cell viability, and suppressed pro-inflammatory cytokine secretion in ARPE-19 and iPSC-derived RPE models exposed to oxidative or metabolic stress [11,15]. These cytoprotective effects are linked to activation of the Nrf2 pathway, which enhances antioxidant enzyme expression and glutathione homeostasis [11]. Given the central role of oxidative damage and chronic inflammation in RPE degeneration, these data provide a plausible explanation for metformin-mediated retinal protection. It may also help explain epidemiologic associations between metformin use and the risk of AMD. However, many in vitro studies used metformin concentrations in the millimolar range (0.5-5 mM), which far exceeded the micromolar plasma levels achieved with oral dosing in humans. This translational gap should be considered when interpreting the relevance of preclinical findings. While the consistency of these findings provides a potential explanation for the positive associations observed in clinical studies, caution is warranted in extrapolating directly to therapeutic dosing.

Mitochondrial dysfunction is a hallmark of AMD, particularly within RPE cells, which are highly metabolically active and exposed to sustained oxidative stress. Across multiple experimental models, metformin demonstrated significant protective effects on mitochondrial structure and bioenergetic function. Notably, iPSC-derived RPE cells from patients with AMD exhibited impaired mitochondrial respiration and structural abnormalities. These were partially reversed by metformin, in addition to RPE-specific functional markers such as PEDF and VEGF secretion [12,13]. These benefits were closely linked to AMPK activation, which promoted mitochondrial biogenesis by increasing the expression of PGC1-α and TFAM. Activation of AMPK also suppressed NF-κB-mediated inflammatory signaling and attenuated EMT, a process associated with RPE dysfunction and degeneration [12].

In addition to metformin’s ability to regulate mitochondrial biogenesis, it can facilitate AMPK activation in RPE cells leading to enhancement of autophagic pathways. This activation promotes clearance of damaged cellular components under environments of oxidative stress [14]. Metformin displaying autophagy-mediated cellular protection highlights AMPK as a potent regulator in RPE cellular health in the context of DNA damage, specifically with ultraviolet radiation exposure [17].

Nuances among the identified data warrant further consideration. Metformin has been shown to exert differing pharmacologic actions. Some studies display activation of the AMPK pathway, while others state it has inhibitory effects on mitochondrial complex I [12,16]. Under certain oxidative stress environments, such as sodium iodate exposure, complex I inhibition exacerbated mitochondrial dysfunction and cytotoxicity, whereas selective AMPK activation preserved mitochondrial respiration and morphology [16]. Overall, these findings indicate that metformin's therapeutic impact in AMD varies depending on the underlying cellular stress and disease context.

Emerging epidemiologic evidence further supports a potential association between metformin use and reduced risk of AMD. Retrospective cohort and case-control studies using large insurance-based databases have suggested that metformin use may be associated with a lower risk of developing AMD in the clinical setting, particularly dry AMD. Observational studies suggested that longer duration of metformin use and higher cumulative exposure may be associated with stronger protective effects against AMD [18-20]. However, these associations do not establish causality and may be influenced by residual confounding factors.

However, as these findings are derived from observational studies, they do not establish a causal relationship between metformin use and AMD risk and may be influenced by residual confounding factors. Nonetheless, the consistency of these associations across studies supports the plausibility of a potential protective relationship. In several analyses, the observed associations persisted after adjustment for diabetes status and related comorbidities, suggesting that the relationship between metformin use and AMD risk may not be solely explained by glycemic control [19].

At the same time, practical considerations should be considered. The observed associations vary based on dosing and duration of treatment, with no optimal treatment window having been identified [16-18,20]. Most existing data are pulled from populations with type 2 diabetes, which may limit applicability to those individuals without diabetes [18-20]. Additionally, despite metformin’s established safety profile, adverse effects such as lactic acidosis [4-8,14] and vitamin B12 deficiency [6,7,9] should be weighed in susceptible patients, such as the elderly or medically frail patients.

Current evidence also supports the potential role of metformin as an adjunctive therapy rather than a stand-alone treatment for AMD. Existing therapies for AMD, particularly anti-VEGF agents, primarily address neovascularization and have a limited impact on oxidative stress and mitochondrial dysfunction [2,3]. Metformin’s ability to target these upstream cellular processes suggests that it may complement existing therapies by modifying disease progression rather than treating late-stage manifestations.

Gaps in knowledge were prevalent throughout the evaluation of the current literature. Specifically, there is no established or direct mechanistic link between metformin and RPE cell telomere preservation. Most studies relied on immortalized or iPSC-derived RPE cells [11-17] with artificial stress conditions [12-16] and utilized metformin concentrations exceeding physiologic levels achievable in humans. Further limitations include clinical studies that are retrospective in nature [18-20], which limit phenotypic precision and adjustment for risk factors such as smoking [19]. Even with these uncertainties, overlapping and promising data from cellular and epidemiologic evidence make a strong case for continued investigation of metformin in AMD.

Based on gaps identified in the current literature, prospective studies are needed to clarify optimal dosing strategies, timing of intervention, and which patient populations with AMD are most likely to benefit from metformin. Integrating advanced retinal imaging and mitochondrial biomarkers into future studies will be beneficial in determining metformin’s role in slowing retinal aging and AMD progression, as well as evaluating its efficacy as a standalone treatment versus in combination with established therapies. Clinical interpretation should also consider limitations related to the generalizability of these findings to broader populations, potential adverse effects such as vitamin B12 deficiency or lactic acidosis in frail patients, and uncertainty regarding how metformin's efficacy compares as a standalone treatment versus in combination with established therapies.

## Conclusions

The current evidence suggests that metformin may offer significant cellular benefits for the RPE through supporting mitochondrial function and reducing stress-related injury. The ability to activate AMPK is a key factor, preserving mitochondrial biogenesis, reducing inflammatory signaling, and preventing maladaptive changes such as EMT. Simultaneously, the ability of metformin to inhibit mitochondrial complex I may reduce metabolic stress under certain conditions. This effect may be harmful in cases of severe mitochondrial dysfunction. These overlapping mechanisms suggest that the benefits of metformin therapy are not due to a single pathway but through a multi-layered metabolic response and overall improvement in mitochondrial stability. Still, most findings come from preclinical research, so these conclusions remain preliminary. Future research, including prospective clinical studies, is needed to better understand how metformin affects cellular health, when it may be beneficial, and whether it could serve as an adjunct treatment for retinal and mitochondrial disease.

## Appendices

To identify relevant studies, we performed systematic searches of Embase, Web of Science, and Ovid MEDLINE for publications between January 2015 and November 2025. Search terms included “metformin” and “biguanides” combined with key mechanistic and AMD-related terms such as “mitochondria,” “mitophagy,” “oxidative stress,” “Nrf2,” “telomere shortening,” “telomerase,” “cellular senescence,” “macular degeneration,” “AMD,” and “retinal pigment epithelium.” The full search strings used for each database are listed below.

A) Embase

('metformin':ti,ab OR 'biguanides'/exp) 

AND 

('mitochondria':ti,ab OR 'mitochondrial function':ti,ab OR 'mitochondrial dysfunction'/exp OR 'mitophagy':ti,ab OR 'oxidative stress'/exp OR 'Nrf2':ti,ab OR 'telomere shortening':ti,ab OR 'telomerase'/exp OR 'cellular senescence'/exp) 

AND 

('age-related macular degeneration'/exp OR 'AMD':ti,ab OR 'retinal pigment epithelium'/exp OR 'RPE':ti,ab)

Total articles retrieved: 17

B) Web of Science

1) TS=(metformin OR biguanides)

AND 

2) TS=(mitochondria OR "mitochondrial function" OR "mitochondrial dysfunction" OR mitophagy 

OR "oxidative stress" OR Nrf2 OR "telomere shortening" OR telomerase OR "cellular senescence")

AND

3) TS=("macular degeneration" OR AMD OR "retinal pigment epithelium" OR RPE)

Total articles retrieved: 22

c) OVID MEDLINE

1) (metformin.ti,ab,kf. OR exp Metformin/ OR exp Biguanides/)

2) (mitochondria.ti,ab,kf. OR "mitochondrial function".ti,ab,kf. 

    OR exp Mitochondria/ OR exp Mitochondrial Dysfunction/ 

    OR mitophagy.ti,ab,kf. OR exp Autophagy/ 

    OR exp Oxidative Stress/ OR Nrf2.ti,ab,kf. 

    OR "telomere shortening".ti,ab,kf. OR exp Telomerase/ 

    OR exp Cellular Senescence/)

3) ("macular degeneration".ti,ab,kf. OR exp Macular Degeneration/ 

    OR AMD.ti,ab,kf. OR "retinal pigment epithelium".ti,ab,kf. 

    OR exp Retinal Pigment Epithelium/)

4) 1 AND 2 AND 3

Total articles retrieved: 7

These searches were conducted in English-language literature and limited to human studies where applicable. The search strategies are provided in full to ensure transparency and reproducibility of the review.
